# The Stigmatisation of Men Who Have Sex With Men in the Spread of Mpox: Are We Repeating the Mistakes of HIV?

**DOI:** 10.1002/nop2.70370

**Published:** 2025-11-04

**Authors:** David Bermejo‐Martínez, Enrique Castro‐Sánchez, Pilar Marqués‐Sánchez

**Affiliations:** ^1^ Department of Nursing and Physiotherapy Campus de Ponferrada S/N, Universidad de León Ponferrada Spain; ^2^ Antimicrobial Innovations Research Centre Brunel University London Uxbridge UK; ^3^ NIHR HPRU in Healthcare‐Associated Infection and Antimicrobial Resistance Imperial College London London UK; ^4^ Research Group on Global Health and Sustainable Human Development University of the Balearic Islands Palma de Mallorca Spain; ^5^ SALBIS Research Group, Department of Nursing and Physiotherapy Campus de Ponferrada S/N, Universidad de León Ponferrada Spain

**Keywords:** HIV, men who have sex with men (MSM), mpox, public health, social determinants of health, stigmatisation

## Abstract

The 2022 mpox outbreak disproportionately affected men who have sex with men (MSM), leading to targeted public health interventions. While data supported prioritising MSM, this focus risks reviving stigmatisation reminiscent of the HIV/AIDS crisis. This editorial examines the roots of this narrative and its potential negative impact on affected communities. We argue that while high‐risk groups should be addressed, public health efforts must avoid reinforcing prejudices and instead adopt inclusive strategies. Expanding prevention beyond MSM, promoting community empowerment, and fostering collaboration are essential to combat both the virus and the stigma it can generate.

In 2022, the outbreak of mpox (formerly monkeypox) triggered a global alert, prompting a public health response that included vaccination and awareness campaigns (Hoxha et al. 2023). The disproportionate incidence of cases reported among men who have sex with men (MSM) fostered a narrative (WHO 2025) with reminiscence to the 1980s, when the gay community was blamed for the spread of the Human Immunodeficiency Virus (HIV).

This editorial explores the roots of this narrative, its potential impact on affected communities, and whether this approach is scientifically valid or merely reinforces old prejudices.

The 2022 mpox outbreak was initially identified in MSM communities in Europe and North America, prompting public health authorities, such as the UK Health Security Agency (UKHSA), to implement measures specifically directed at this population (UK Health Security Agency 2024a). For example, by August 2022, more than 70% of reported cases in England involved MSM, and thus vaccination criteria focused on this group, particularly those individualsengaging in high‐risk sexual behaviours, such as having multiple sexual partners, participating in group sex, or attending venues where sexual activity takes place (UK Health Security Agency 2024b).

By mid‐2024 the European Centre for Disease Prevention and Control (ECDC) reported that 98% of Mpox cases in Europe were among MSM (Centre for Disease Prevention E 2024). While the decision to initially prioritise vaccination within this community appears to be supported by epidemiological data, the persistent focus on MSM may have rekindled the stigma that spread during the HIV epidemic. During that crisis, the media and some public health authorities unfairly blamed MSM for the spread of the virus, leading to a surge in homophobia (Keum et al. 2023).

Would the epidemiological perspective truly be the sole reason for this emphasis on MSM, or might old biases and prejudices be also at play, risking the enactment of past mistakes and their consequences? In the case of HIV, the stigmatisation of MSM had devastating effects, such as creating organisational and psychological barriers to accessing social and healthcare systems. According to the US National Institute of Allergy and Infectious Diseases (NIAID), this discrimination not only caused unnecessary suffering but also hindered public health efforts to contain the spread of HIV (Final IAS 2023 Conversations 2024), and likely discouraged MSM from engaging in other initiatives developed by health systems (Jennings et al. 2019).

Anyone, regardless of their sexual orientation, can contract mpox. The virus is primarily transmitted through close contact, including sexual, but also through other close physical interaction (Brainard et al. 2024). Therefore, singling out MSM as mainly responsible for the transmission of mpox would be, at the very least, an oversimplification.

The stigmatisation of MSM in the emerging mpox narrative reflects a growing and concerning trend of using epidemics, their determinants, and the effects of containment measures implemented as a pretext to perpetuate social prejudices (Saeed et al. 2020). Labeling a disease as exclusive or predominantly linked to a specific community renders other affected groups invisible and may foster a false sense of security among those outside that community. Public health agencies have a challenging responsibility, balancing data‐informed communication with the consideration of the social and psychological impacts of their institutional messages. While initial data pointed unquestionably to a higher prevalence of cases among MSM, it is crucial that communication strategies avoid the trap of associating a disease with a particular group, especially when that group is already marginalised and vilified.

In this regard, expanding vaccination efforts to include more individuals, such as sex workers and other groups at high risk of infection, is a step in the right direction. This more inclusive approach can help curb virus transmission as well as harmful stigmas (Brainard et al. 2024).

An important lesson learned from the HIV epidemic was the vital contribution of community empowerment to clinical, public health, and rights maintenance and recovery success. Instead of focusing on implicitly blaming MSM for the continued dissemination of mpox, health authorities should work closely with these communities to promote prevention and access to vaccination. Additionally, it would be beneficial to recognise the significance of social determinants of infection, considering the intersectionality of these determinants and avoiding placing the burden of success of public health campaigns solely on the shoulders of the most vulnerable individuals. Instead, the responsibility towards this success should be distributed across the network of power structures that underlie these issues.

As highlighted by the *British Medical Journal* (BMJ), many sexual health clinics in the UK reported a shortage of vaccines alongside high demand for them, suggesting that despite barriers, there was a significant level of awareness and willingness to self‐protect within the MSM community (Dye and Kraemer 2022). Ultimately, stigmatisation is not only unjust but also counterproductive to public health efforts (Makurumidze et al. 2022).

The dynamism and flexibility of health campaigns and interventions should reflect and respond to the realities described by epidemiology, especially following the initial stages of the mpox outbreak. The key to combating this emerging infection in our context lies in an inclusive response that, at the very least, does not disempower the affected communities but instead harnesses their resilience and self‐efficacy, fostering solidarity rather than division.

Nurses play a critical role in addressing the mpox epidemic through direct care, community engagement, and public health advocacy. They are at the frontline of education, helping to dispel myths and combat stigmatisation while empowering vulnerable populations. Nurses are also uniquely positioned to promote inclusive vaccination campaigns and provide culturally competent care, ensuring that affected individuals feel supported and respected. Furthermore, as trusted healthcare professionals, nurses can act as advocates for equitable healthcare policies, emphasising the importance of addressing the intersectionality of social determinants of health to achieve better outcomes for all. By fostering trust and inclusivity, nurses contribute not only to clinical success but also to the reduction of stigma and the promotion of public health solidarity.

## Author Contributions

All authors have actively contributed to each stage of the manuscript preparation process, from initial conception and design, drafting, and final revisions.

## Conflicts of Interest

The authors declare no conflicts of interest.

## Data Availability

The authors have nothing to report.
